# Experimental Data and Thermodynamic Modeling of Fructose Solubility in Glycerol

**DOI:** 10.1021/acsomega.4c08835

**Published:** 2025-03-14

**Authors:** Lucas H. J. Morita, Vitor H. Ferreira, Carlos E. Crestani

**Affiliations:** Federal Institute of Education, Science and Technology of São Paulo (IFSP), R. Stefano D’Avassi, 625, Matao, São Paulo 15991-502, Brazil

## Abstract

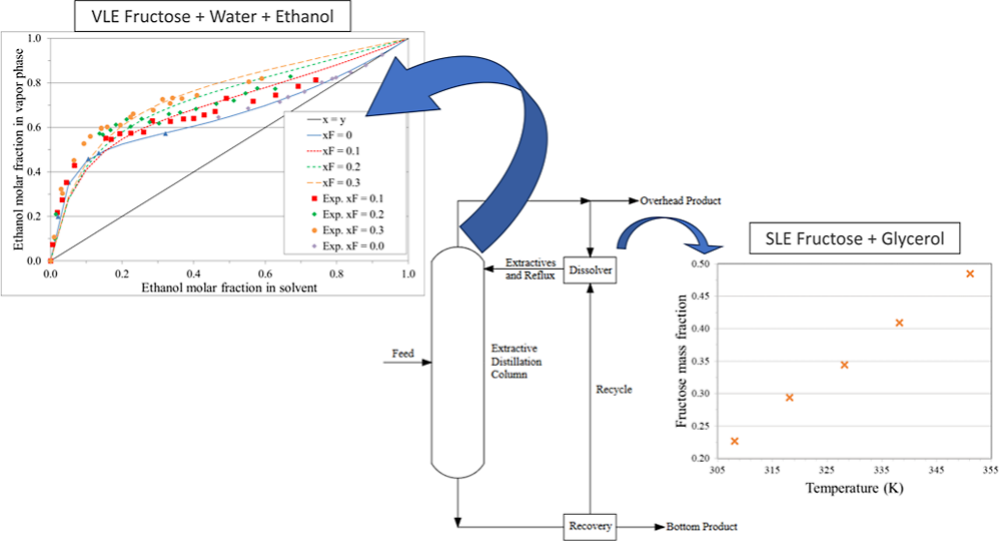

Extractive distillation is widely used in industries such as anhydrous ethanol manufacturing. History shows several problems related to separation agents, such as chloroform, cyclohexane, ethyl ether, carbon tetrachloride, and ethylene acetate. Environmental agencies have restricted the use of several solvents. There is an opening for research into less toxic and more effective dehydrating agents. Both glycerol and fructose are potential separation agents; glycerol has not proven viable for commercial operation to date, and fructose, despite its potential demonstrated in the literature, has the limitation of adding a solid to the top of a distillation column. Hence, glycerol is intended to add fructose to the extractive distillation column, which makes it necessary to know the solubility of fructose in glycerol. This work addresses new experimental data on fructose solubility in glycerol for temperatures ranging from (308.15 to 351.15) K, together with a fit with Nývlt Equation and thermodynamic modeling of the equilibrium, which are essential for predicting the quaternary equilibrium of water, ethanol, fructose, and glycerol. The study of extractive distillation for producing anhydrous ethanol using glycerol and fructose as extracting agents has great potential for application in the industry, as demonstrated by the studies carried out so far.

## Introduction

Due to climate issues and dwindling oil, the world seeks renewable energy. Brazil has been investing more in biofuels to meet global agreements. It is a leading producer of ethanol (production of 2.16 billion liters of hydrated ethanol is estimated in the first half of 2024, an increase of 13.3% compared to the same period last year^1^), and its production of other biofuels, like biodiesel and biokerosene for aviation, is growing.

The production of ethanol from sugar cane involves several key steps: the sugar cane is processed to extract its juice, which is treated in a physical-chemical treatment that involves filtration, addition of chemicals, and decantation. This clarified broth is used to prepare the must that will be fermented, converting sucrose into ethanol through a biochemical pathway. The resulting product is then purified through distillation. Standard distillation can purify ethanol up to 96 °GL or hydrated ethanol. However, at this point, an azeotrope is formed, where the composition of the liquid and vapor phases is the same, making separation by simple distillation impossible.^2^

This hydrated ethanol, used as fuel, must have an alcohol content between 92.5% and 94.6% w/w.^3^ However, ethanol dehydration must be added to use it as an additive to gasoline; conventional distillation cannot be used, as ethanol and water form an azeotropic mixture. Some distillation processes have been developed to overcome this difficulty; a third compound is added to the mixture in azeotropic,^4^ extractive,^5^ or reactive distillation. There are also processes using membranes, adsorption, and diffusion^6^ that are more complex, generally not directly viable, but do not require adding other components to the process. In this sense, there are works focused on analyzing distillation and pervaporation processes based on the overall energy requirement, consumption, and economics.^7^

Anhydrous ethanol, in addition to being used as biofuel, is also used as a raw material in the production of esters and ethers and as a solvent in the manufacture of paints, medicines, and food.^8^ In recent years, other alternatives have been studied, such as molecular simulations of zeolite nanosheets as reverse osmosis (RO) membranes in ethanol/water^9^ and performance analysis of these processes;^10^ however, estimated required energy on this processes are more significant than on distillation.

Extractive distillation is one of the most widely used processes in industries for separating azeotropes, including producing anhydrous ethanol.^11^ The history of the sector shows several problems related to agents commonly used in the process, such as chloroform (toxic to living beings in general), cyclohexane (fatal if ingested or enters respiratory tract), ethyl ether (inhalation causes drowsiness and dizziness), carbon tetrachloride (depression of the central nervous system), and ethylene acetate (respiratory irritation). For this reason, environmental agencies have restricted the use of several solvents. In the Brazilian production of absolute ethanol, 70% of the plants use cyclohexane, which, although permitted, can also cause serious harm to human health, in addition to being extremely flammable. In this context, Soares^12^ says there is an opening for research into other dehydrating agents that are less toxic and more effective, which contributes to making the processes more sustainable. Among the potential dehydrating agents for ethanol, some are solid. In extractive distillation, while solids need to be dissolved in a liquid phase at a point near the top of the column, liquid agents are more accessible to transport, handle, and mix, justifying the greater demand for them in the past.^13^ A solid to be added to the top of the column needs to be soluble in the less volatile component and very slightly soluble in the more volatile component; it needs to be introduced at a constant rate without vapor escaping, which could generate losses and clogging.^14^

Some salts and sugars can be cited as potential solid-extracting agents. Fructose is mentioned in the literature as a possible agent for breaking the ethanol/water azeotrope; it is a sugar originating from the inversion of sucrose and, therefore, present in the sugar-energy industry, both as syrup and with possibilities for producing its crystalline form.^15^ Salts such as sodium chloride (one of the most essential and well-known salts in inorganic chemistry), calcium chloride (produced from limestone, used in refrigeration machines, dust, and ice control on roads, cheese, and cement) and sodium acetate^12^ are also cited as potential agents for this separation.

One possibility for studying such solids is using a solvent to dissolve them. Glycerol as a solvent is desirable in the Brazilian context since it is renewable and a byproduct of biodiesel production.^16,17^ It is a green solvent,^18^ a low-cost alternative that contributes to reducing the environmental impact of the process; in addition, there are results in the literature showing its potential as a separation agent, although alone, it is not yet a viable alternative to the process. Dissolving a solid separation agent could increase its separation potential and make its use feasible. Matugi^19^ conducted a theoretical study on using salts dissolved in glycerol, showing potential results that still require experimental validation.

Therefore, it is necessary to dissolve it in a solvent to consider the application of fructose as a separation agent in the production of anhydrous ethanol. Studying this solubility is the first step in such research. Segur and Miner^20^ studied the solubility of sucrose and dextrose in glycerol in the past, but the solubility of fructose in glycerol (as well as the salts mentioned above) is unknown. In this context, the present work aimed to determine the solubility of fructose in glycerol at temperatures close to those of the operation of ethanol distillation columns.

A possibility that may be of interest to the industry is the mathematical modeling of the vapor–liquid equilibrium (VLE) of the quaternary mixture composed of fructose, glycerol, ethanol, and water. This study would show the way for the next experimental step in the study of this solution. Data on the phase equilibrium of solutions containing sugars are limited, especially regarding nonalcoholic solvents; if practical, thermodynamic modeling would benefit this work. Thermodynamically modeling systems containing sugars is not a simple task due to their complex behavior in solution, either due to the chemical equilibrium of their tautomers or the associative forces generated by the hydroxyls present in the solution. However, if the modeling is effective, it can allow testing and optimization of processes such as extractive distillation to produce anhydrous ethanol, as mentioned above.

In this sense, the present work chose some of the thermodynamic methods available in the literature for determining the phase equilibrium of solutions containing sugars, which will be evaluated by comparing them to the experimental data on the solubility of fructose in glycerol obtained. This comparison aims to assess their initial feasibility in dealing with solid–liquid equilibrium (SLE) and, in a second step, their application in studying the quaternary VLE of fructose, glycerol, ethanol, and water. The generic Nývlt equation for determining solubility^21^ and four predictive methods based on group decomposition for calculating activity coefficients were used in this study: the method proposed by Kuramochi et al.,^22^ known as Bio-UNIFAC; P&M-UNIFAC^23^ which uses the groups created by Catté et al.;^24^ A-UNIFAC^25^ which, in addition to using the groups created by Catté et al., added to the traditional UNIFAC equation the associative contribution, referring to the interactions between the hydroxyls present in solution and, finally; a modification of the method proposed by Spiliotis and Tassios,^26^ known as mS-UNIFAC.^27^ The suggestion of including the OHgly subgroup with the interaction parameters was also tested;^28^ this application was performed in the A-UNIFAC model (with the authors’ original OHring group for the fructose hydroxyls).

## Experimental Section

### Chemicals

Commercial solid fructose (1,3,4,5,6-pentahydroxyhex-2-one) P.A. (Synth), with a mass fraction purity of 0.9949, and anhydrous glycerol 1,2,3-propanotriol P.A. (Synth), a pharmaceutical primary standard, with 0.995 mass fraction purity, were used in experiments without additional purification steps, as Table 1 shows.

**Table 1:** Sample Table

chemical name	CAS Reg. no	suppliers	initial mass fraction purity	purification method	final mass fraction purity	analysis method
fructose^a^	57-48-7	Synth	0.9949	none		
glycerol^b^	56-81-5	Synth	0.995	none		

a 1,3,4,5,6-Pentahydroxyhex-2-one.

b Propane-1,2,3-triol.

### Equipment and Glassware

Shaker SOLAB model SL-222 with temperature control (error of 0.1 °C), analytical balance (error of 0.0001 g), mercury thermometer (error of 0.11 °C), and 250 mL Erlenmeyer flask with perforated stopper for thermometer.

### Experimental Procedure

The method used to determine fructose solubility in glycerol as a solvent is based on the one proposed by Myerson,^29^ with some adaptations. The procedure was repeated for constant temperatures of 308.15, 318.15, 328.15, 338.15, and 351.15 K (the maximum temperature of the equipment), which is why the method is called isothermal.^29^

In a shaker with temperature control (error of 0.1 °C), glycerol samples were added in triplicate with a known quantity of fructose, initially based on the solubility of sucrose in glycerol, as presented by Segur and Miner.^20^ A thermometer was placed inside the Erlenmeyer flasks containing the solutions to ensure the temperature and correct the shaker’s temperature set point, if necessary. For the tests at a temperature of 308.15 K, 165 g of glycerol and 35 g of fructose were initially added to each of the 3 Erlenmeyer flasks; for the tests at 318.15 K, 152 and 48 g; for the tests at 328.15 K, 134 and 66 g; for the tests at 338.15 K, 119 and 81 g; and for the tests at a temperature of 351.15 K, 137 and 63 g of glycerol and fructose, respectively.

The solutions were kept under agitation at the constant temperature of the experiment, and every 24 h, the solution was visually evaluated. If no crystals were present, 1 g of fructose was added and kept under agitation for another 24 h. The procedure was repeated until the fructose had not completely dissolved in the glycerol—at this point, an additional 24 h was given to the suspensions. The agitation was maintained at 300 rpm (a previous test was performed to ensure the suspension of fructose in glycerol).

### Thermodynamic Modeling

Methods for describing the phase equilibrium of solutions containing sugars have emerged since the 1990s. The methods can be based on adjustments to experimental data of the solution to be studied or can be predictive, such as those based on the UNIFAC method. An equation widely used to adjust solid–liquid equilibrium data is the generic solubility equation proposed by Nývlt.^30^ This equation has three adjustable parameters available in the literature for several substances. Another widely used equation was proposed by Peres and Macedo^31^ and is based on adjusting the interaction parameters of the UNIQUAC method with a modification in calculating the combinatorial contribution proposed by Larsen et al.^32^ The calculation method is known as P&M-UNIQUAC; both require specific data from the solution under study.

When specific experimental data from the solution to be worked on are unavailable, an alternative is to use predictive methods. Several methods in the literature are based on the traditional UNIFAC method and the modified equation.^32^ Regarding the prediction of equilibrium data for solutions containing sugars, the methods are generally based on creating new functional groups to represent the behavior of the molecules in solution without completely dissociating the sugar molecule due to the proximity of the functional groups found in it. Interactions between groups are essential for determining the interactions of the component, and the creation of new groups also allows the differentiation of isomers, such as glucose and fructose.

Abed et al.,^33^ Catté et al.,^34^ Kuramochi et al.,^22^ and Spiliotis & Tassios^35^ are examples of authors who created new decomposition groups to represent sugar molecules. However, the use of these methods in solutions containing alcohols must be previously evaluated due to the interactions between the hydroxyl groups (OH) present in the solution, which can influence the quality of the model adjustments. More recently, many applications of UNIFAC-based methods have been found in mixtures from biodiesel production, mainly in the study of LLE (liquid–liquid equilibrium). The presence of hydroxyls in these mixtures has been subject to modifications such as the inclusion of ethanol and methanol as UNIFAC subgroups in specific mixtures;^36,37^ Bacicheti et al.^38^ used EtOH-B, focused on biodiesel production, and EtOH-D focused on the deacidification process, for an example. Rios et al.^39^ studied biodiesel blends using the UNIQUAC method for oils from a specific biodiesel manufacturing feedstock. There are also other studies involving the LLE equilibrium of biodiesel blends, that of petroleum fuels^40−42^ and also in determining the physicochemical properties of ecotoxicants.^43^

The applications of thermodynamic modeling based on UNIFAC methods show the practical relevance of these theoretical studies; the A-UNIFAC model is widely cited in the literature in articles related to the food industry and in the calculation of thermodynamic properties of compounds of interest.^44,45^ Kuramochi et al. applied their Bio-UNIFAC model to predict VLE and LLE relevant to crude biodiesel fuel’s separation and purification processes in binary and ternary mixtures.^46^

The generic Nývlt equation for determining solubility and four predictive methods were used for mathematical modeling: Bio-UNIFAC, P&M-UNIFAC, A-UNIFAC, and mS-UNIFAC. The suggestion of including the OHgly subgroup with the interaction parameters obtained by Bessa et al.^47^ for the hydroxyls present in glycerol in the A-UNIFAC model (with the authors’ original OHring group for the fructose hydroxyls) was also tested, resulting in better results than the original model. The models were chosen based on a literature review of their development and use. The models chosen are those that present good correlations with experimental data from solutions containing fructose (all models developed to model solutions with sugars) and other solvents other than water, whether in solid–liquid or liquid–vapor equilibrium (since this will be the final objective of the study in extractive distillation).

As thermodynamic models based on the calculation of the activity coefficient were used, the solid–liquid equilibrium calculations were performed using the expression of eq 1(48)
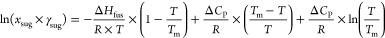
1in this equation, *x*_sug_ is the sugar solubility at the solution temperature (*T*) expressed in molar fraction; γ_sug_ is the activity coefficient of sugar in solution calculated by the thermodynamic model; Δ*H*_fus_ is the melting enthalpy at the normal melting temperature (*T*_m_), and Δ*C*_p_ is the difference between the heat capacity of pure solvent and solid sugar; it is assumed to be not dependent on temperature. The values of the physical properties of fructose at ambient pressure are found in Table 2.

**Table 2:** Fructose Physical Properties of Fructose at 760 mmHg^a^

property	value	unity
Δ*H*_fus_	26,030^49^	J·mol^–1^
*T*_m_	376.15^50^	K
*C*_P_^S^	232^50^	J·mol^–1^·K^–1^

a Δ*H*_fus_ is the melting enthalpy at the normal melting temperature (*T*_m_), and Δ*C*_p_ is the difference between the heat capacity of pure solvent and solid sugar.

The parameters *R*_k_ and *Q*_k_ of glycerol for relative molecular volume (*r*) and relative molecular surface area (*q*) calculation were those of Rostami et al.^51^ All calculations were performed in Microsoft Excel spreadsheets validated with classic examples of calculations via UNIFAC available in Poling, Prausnitz, and O’Connel^48^ and calculations of other works of the group.^15,52,53^

## Results and Discussion

### Experimental Results

Table 3 shows the solubility of fructose in glycerol at temperatures between 308.15 and 351.15 K and the average deviation (σ). The deviations were not significant in any temperature range, even though the viscosity of the solution varied as a function of temperature. High viscosities can generate experimental problems, mainly if the procedures include sample extraction, filtration, etc., which was not the case. Glycerol and its solutions are known for their considerable viscosity, especially at temperatures higher than 333.15 K.^54^ In this sense, the assembled system, with the Shaker agitated at 300 rpm, proved to be quite functional for the purpose and the possible problems.

**Table 3:** Average of Experimental Fructose Solubility (Fructose Mass Fraction *x*_F_) in Glycerol at Different Temperatures *T*, and the Average Deviation, σ^a^

T/K	*x*_F_	σ
308.15	0.2270	0.0036
318.15	0.2942	0.0030
328.15	0.3440	0.0038
338.15	0.4089	0.0043
351.15	0.4734	0.0099

a Standard uncertainties are *u*(*T*) = 0.01 K and *u*(*x*_F_) = 0.0002.

To give a perspective of the solubility of the sugars fructose, glucose, and sucrose in glycerol as a solvent, Table 4 presents the average values obtained for fructose solubility at 308.15 K together with that of Segur and Miner^20^ for glucose and sucrose at this same temperature, the authors conducted their experiments from (288.15 to 308.15) K.

**Table 4:** Solubility of Sugars in Glycerol

sugar	solvent	solubility	unit
glucose	95% glycerol	22.2^20^	g/100 mL
sucrose	95% glycerol	17.3^20^	g/100 mL
fructose	100% glycerol	22.6973^a^	g/100 g

a This work.

The solubility of sucrose in water at 308.15 K is higher than that of glucose in water, and both are lower than that of fructose in water.^23^ In solutions containing glycerol as the main solvent, glucose was more soluble than sucrose in 95% glycerol and 5% water −22.2 g/100 mL. The solubility of fructose in glycerol (100%) at 308.15 K obtained in this work was 17.9594 g/100 mL. Furthermore, the behavior of the solution shows an increase in the solubility of fructose in glycerol with increasing temperature, which is common in solutions with sugars that have high variations in dissolution enthalpy. The highest solubility value was obtained at the highest temperature in the present study, 351.15 K, the boiling temperature of anhydrous ethanol. With a higher solubility at this temperature, the potential for application of this mixture as a separation agent in an extractive distillation for the production of anhydrous ethanol is even more significant since thermodynamic studies show that the greater the amount of dissolved fructose, the better the ethanol–water separation.^55^

### Mathematical Modeling Results

**Figure 1:**
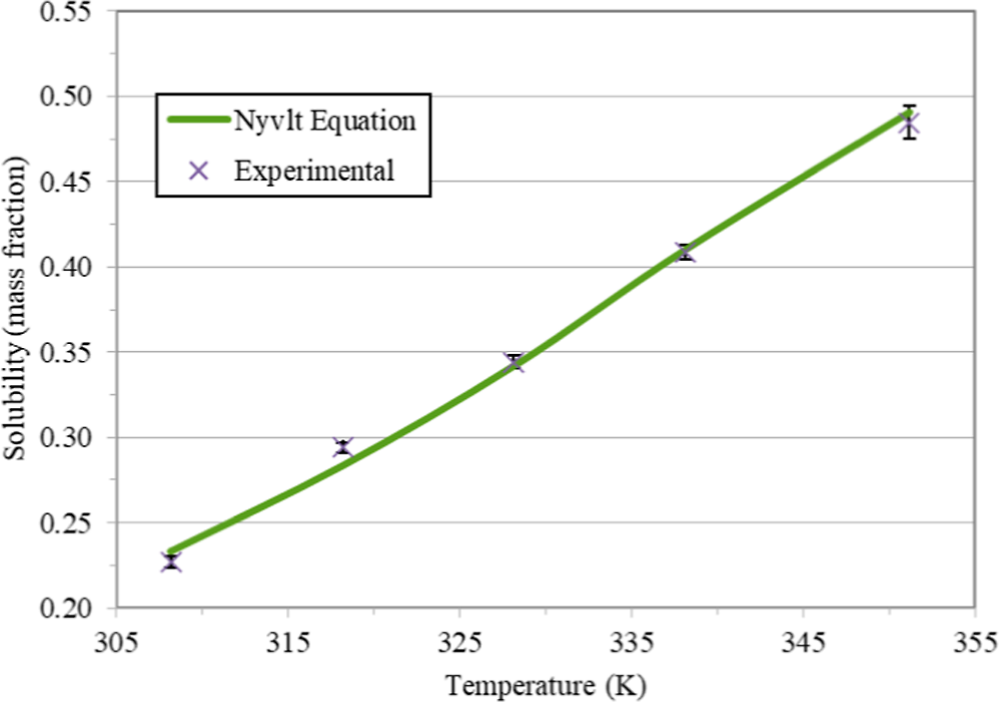
Fructose solubility in glycerol expressed as mass fraction and adjustment with Nývlt equation.

**Figure 2:**
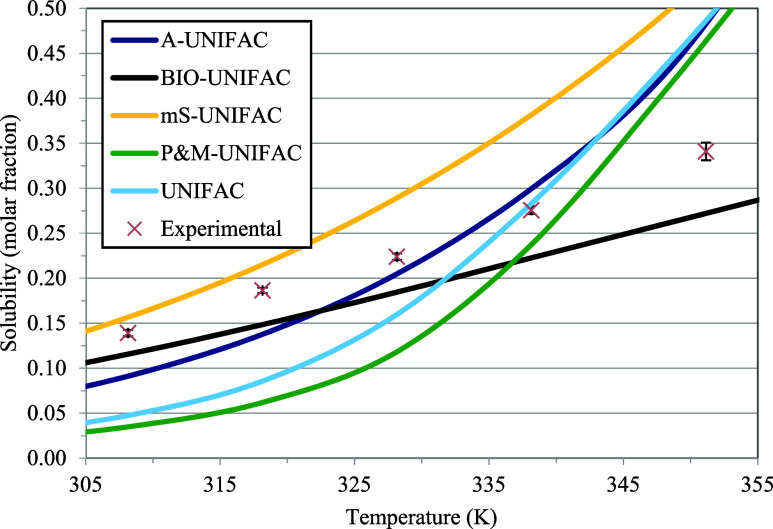
Fructose solubility in glycerol expressed as a molar fraction. Comparison among experimental data and different thermodynamic models.

Figures 1 and 2 present the experimental solubility of fructose in glycerol as a function of temperature and data calculated by the Nývlt solubility equation, the traditional UNIFAC decomposition method, and the UNIFAC-based methods: A-UNIFAC, Bio-UNIFAC, mS-UNIFAC, and P&M-UNIFAC. Table 5 presents the root mean square deviations (RMSD) between experimental solubility data and calculated data from the models UNIFAC, A-UNIFAC, Bio-UNIFAC, mS-UNIFAC, and P&M-UNIFAC.

**Table 5:** Root Mean Square Deviations (RMSD %) of Thermodynamic Models Compared to Experimental Data

model	RMSD
A-UNIFAC	22.47%
Bio-UNIFAC	18.58%
mS-UNIFAC	26.11%
P&M-UNIFAC	55.92%
UNIFAC	42.15%

Equation 2 presents the solubility model proposed by Nývlt, with the parameters adjusted for this work’s experimental data.
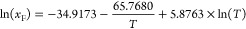
2

In this equation, *x*_F_ is the fructose solubility expressed in mass fraction at the solution temperature (*T*) expressed in *Kelvin*. The first two terms on the right-hand side of the equation relate to the variation of the activity coefficient, and the last term is related to the effect of temperature on the enthalpy of fusion. The fit to the Nývlt solubility equation results in a RMSD of 0.96%, making it suitable for calculating solubility within the temperature range of the experimental data. Since it is a fit for the experimental data, this mathematical model cannot be used outside this range.

The UNIFAC method is a thermodynamic calculation method based on estimating activity coefficients, which can be used as a predictive method. The method is based on the decomposition of molecules in solution into groups, and the interaction between these groups, obtained experimentally from a type of phase equilibrium, can be used to calculate a phase equilibrium that does not have experimental data but involves the same groups and subgroups as the initial estimate.

In general, none of the models correlated well with the experimental data. The P&M-UNIFAC method, which was developed from phase equilibrium (SLE and VLE) of aqueous solutions of various sugars, was used here predictively with glycerol as the solvent. The method was previously tested for solutions containing ethanol, water, and fructose, and even at temperatures outside the range of development of the method, it presented promising results for estimating SLE.^52^ In the present work, with an average deviation of more than 50%, it was not shown to be accurate in calculating the solubility of fructose in glycerol. The same can be said about using the traditional UNIFAC; with high RMSD, which proved unfeasible for direct use in this calculation. The Bio-UNIFAC method, on the other hand, was developed using a much larger number of biochemicals, including the use of equilibrium conditions containing electrolytes. The equilibrium studied in this work presented deviations of less than 20%, which are still considered too high to allow its use. The A-UNIFAC and mS-UNIFAC methods have presented the best results in literature in solutions containing fructose (and ethanol).^52,55,56^ In this work, the A-UNIFAC method, even with the inclusion of OHgly to represent hydroxyls in glycerol, resulted in an RMSD of 22.47%. For the studied solution, the mS-UNIFAC method presented an error of 26.11%. When this method was applied to the solubility of fructose in water and ethanol, the mathematical model presented promising results in solutions with 60% by mass of ethanol in the solvent; for larger values, its deviations were close to 10%.^52^ Even so, when this model was used to estimate the VLE data of aqueous solutions containing fructose and ethanol and the modeling results were good, with deviations of less than 3.6%.^55^ It can be expected that VLEs can be better modeled with these equations and parameter sets, but modifications and new parameter estimates may be necessary with VLE data, mainly from glycerol-containing solutions.

This work presents experimental and mathematical modeling data on the solid–liquid equilibrium of fructose in glycerol. With these data in hand, the next step is to begin studies, either experimental or mathematical modeling, on the application of this solution as a separation agent for the ethanol–water mixture via extractive distillation, which results in a quaternary glycerol-fructose-ethanol–water mixture to be studied. The results of this work show that fructose solutions in glycerol with maximum concentrations of 22% to 48% by mass can be studied, depending on the study temperature. Mathematical modeling of pure fructose in the ethanol–water binary VLE shows that the higher the fructose concentration, the better the ethanol/water separation; however, with it dissolved in glycerol, also a separation agent, this concentration can be a study parameter.

## Conclusions

The solubility of fructose in glycerol was determined experimentally at temperatures between 308.15 and 351.15 K. Data on the solubility of fructose in glycerol are not available in the literature and are very useful in the study of extractive distillation for the production of anhydrous ethanol, which can be performed with fructose as an extracting agent, based on the existence of these data. The experimental procedure was repeated three times for all experimental points, and the average values were presented as the result, with errors small enough to validate the methodology used, in the authors’ view. The results show an increase in fructose solubility with temperature, with the highest solubility obtained at the highest temperature studied in this work, 351.15 K. The experimental data could be adjusted by the Nývlt equation of solubility, with a deviation of 0.96%, which allows the calculation of the solubility of fructose in glycerol with eq 2 for any temperature value between 308.15 and 351.15 K.

Models based on the calculation of the activity coefficients were tested comparatively and compared with the experimental data as methods for predicting the solid–liquid equilibrium of the studied solution. The Bio-UNIFAC method was the one that best predicted the SLE values with a high deviation of 18%. Using models based on UNIQUAC was impossible because there was no data on the glycerol/fructose interaction. The models tested have demonstrated a good ability to predict the equilibrium of solutions containing fructose and solvents such as water and ethanol. Therefore, they are still potential methods for predicting the quaternary equilibrium of water, ethanol, fructose, and glycerol, aiming the study of extractive distillation for the production of anhydrous ethanol using glycerol and fructose as extracting agents, a study with great potential for application in the industry, as demonstrated by the studies carried out so far.

This work fills a gap in the literature regarding experimental data on fructose in glycerol, which were previously nonexistent to the best of the authors’ knowledge. This work also presents mathematical methods commonly used to determine solid–liquid and liquid–vapor equilibrium of solutions containing sugars, showing a low correlation when glycerol is the solvent under study; the use of these models with glycerol was also unprecedented in the literature to the best of our knowledge. On the other hand, concerning mathematical modeling, the Nývlt Equation adjusted the experimental data accurately. It can be used, with the coefficients presented in this work, to determine the solubility of fructose in glycerol at any temperature between 308.15 and 351.15 K.

## Supporting Information Available

Supporting Information presents the complete set of experimental data: triplicates 1, 2, and 3 of fructose solubility in glycerol at different temperatures, the average solubility, and the average deviation (PDF)
