# Supplementary material for: Experimental Data and Thermodynamic Modeling of Fructose Solubility in Glycerol
Source: ACS Omega. 2025 Mar 14;10(11):10911–7. doi: 10.1021/acsomega.4c08835 (PMC11947774; doi:10.1021/acsomega.4c08835)
Supplement: Supplementary file 1 — ao4c08835_si_001.pdf [file ao4c08835_si_001.pdf]

Experimental data and thermodynamic modeling of fructose solubility in glycerol

*Lucas H. J. Morita, Vitor Hugo Ferreira, Carlos E. Crestani**

Federal Institute of Education, Science and Technology of São Paulo (IFSP), R. Stefano D'Avassi, 625, Matao, SP, 15991-502, Brazil.

* Corresponding author: Carlos Eduardo Crestani, Federal Institute of Education, Science e Technology of Sao Paulo – IFSP, Stéfano D´Avassi, 625 - Nova Cidade, 15991-502, Matao, SP, Brazil. E-mail address: cecrestani@ifsp.edu.br, tel. +55 16 98100-9508, correspondence author.

Supplementary material

Table S1. Experimental fructose solubility (fructose mass fraction x_F) in glycerol at different temperatures T: Triplicates 1, 2 and 3, Average solubility and the Average Deviation, σ

T (K)	x_F				σ
	1	2	3	Average	
308.15	0.2276	0.2198	0.2335	0.2270	0.0036
318.15	0.2925	0.2976	0.2924	0.2942	0.0030
328.15	0.3437	0.3405	0.3480	0.3440	0.0038
338.15	0.4139	0.4068	0.4060	0.4089	0.0043
351.15	0.4668	0.4687	0.4847	0.4734	0.0099

Standard uncertainties are $u(T)$ = 0.01 K and $u(x_F)$ = 0.0002
